# A frailty index from common clinical and laboratory tests predicts increased risk of death across the life course

**DOI:** 10.1007/s11357-017-9993-7

**Published:** 2017-09-02

**Authors:** Joanna M. Blodgett, Olga Theou, Susan E. Howlett, Kenneth Rockwood

**Affiliations:** 1 Department of Medicine, Dalhousie University, 5955 Veterans’ Memorial Lane, Halifax, Nova Scotia Canada; 2 MRC Unit for Lifelong Health and Ageing, UCL, London, UK; 3 Departments of Pharmacology and Medicine (Division of Geriatric Medicine), Dalhousie University, Halifax, Nova Scotia Canada

**Keywords:** Frail elderly, Frailty, Deficit accumulation, Biomarkers, Ageing, NHANES

## Abstract

A frailty index (FI) based entirely on common clinical and laboratory tests might offer scientific advantages in understanding ageing and pragmatic advantages in screening. Our main objective was to compare an FI based on common laboratory tests with an FI based on self-reported data; we additionally investigated if the combination of subclinical deficits with clinical ones increased the ability of the FI to predict mortality. In this secondary analysis of the 2003–2004 and 2005–2006 National Health and Nutrition Examination Survey data, 8888 individuals aged 20+ were evaluated. Three FIs were constructed: a 36-item FI using self-reported questionnaire data (FI-Self-report); a 32-item FI using data from laboratory test values plus pulse and blood pressure measures (FI-Lab); and a 68-item FI that combined all items from each index (FI-Combined). The mean FI-Lab score was 0.15 ± 0.09, the FI-Self-report was 0.11 ± 0.11 and FI-Combined was 0.13 ± 0.08. Each index showed some typical FI characteristics (skewed distribution with long right tail, non-linear increase with age). Even so, there were fewer people with low frailty levels and a slower increase with age for the FI-Lab compared to the FI-Self-report. Higher frailty level was associated with higher risk of death, although it was strongest at older ages. Both FI-Lab and FI-Self-report remained significant in a combined model predicting death. The FI-Lab was feasible and valid, demonstrating that even subclinical deficit accumulation increased mortality risk. This suggests that deficit accumulation, from the subcellular to the clinically visible is a useful construct that may advance our understanding of the ageing process.

**Electronic supplementary material:**

The online version of this article (10.1007/s11357-017-9993-7) contains supplementary material, which is available to authorized users.

## Background

As people age, they are more likely to die, but not everyone at the same age has the same risk of death. This unmeasured heterogeneity in the risk of death was termed “frailty” in a *Demography* report (Vaupel et al. 1979). There, it was seen as a fixed factor, present across the life course. Variability in mortality occurs because, despite characteristic age-related changes across the life course (Kim et al. 2017; Mitnitski and Rockwood 2016), ageing occurs at different rates in both humans (Canevelli et al. 2017; Kim et al. 2013; Kulminski et al. 2016; Mitniski et al. 2013; Mitnitski et al. 2017; Rockwood et al. 2017; Whitehead et al. 2014) and in animals (Feridooni et al. 2014; Miller 2017; Parks et al. 2012; Whitehead et al. 2014).

The *Demography* report was notably silent on how that frailty might manifest in individuals. Although there are now hundreds of operational definitions of frailty, since 2001, two general views have emerged. One view sees frailty as a clinical syndrome or *phenotype* (Fried et al. 2001). Another view—from our group—sees frailty as a state of impaired health arising from the accumulation of health deficits (Mitnitski et al. 2001). Health deficit accumulation is usually operationalized in a frailty index (FI), in which deficits can be considered by any set of symptoms, signs, medical illnesses, low self-rated health and specific “geriatric giant” conditions as polypharmacy, cognitive impairment, functional impairment, poor mobility and balance. One advantage, exploited here, is that the FI allows us to quantify how variability in ageing might arise across the adult life course (Rockwood et al. 2011, 2017).

The FI operationalizes frailty as the proportion of health deficits present in a given individual; as the FI score increases, so does the risk of adverse health outcomes, including death (Blodgett et al. 2016; Feridooni et al. 2014; Howlett and Rockwood 2013; Howlett et al. 2014; Klausen et al. 2017; Miller 2017; Rockwood et al. 2017; Whitehead et al. 2014). By this account, the reason that people (or even animals) of the same age have varying risk is that they have varying numbers of health deficits (Mitnitski et al. 2002; Rockwood et al. 2017). In consequence, understanding heterogeneity in ageing in relation to deficit accumulation offers an opportunity to better understand ageing itself (Maffei et al. 2017; Mitnitski et al. 2016, 2017).

Ageing itself is a property of a system. It arises from an interaction of elements at cellular, tissue and organ levels (Kirkwood 2011; Mitnitski et al. 2017). How these ageing phenomena at cellular, tissue and organ levels scale up to become clinically detectable is not yet clear (Howlett and Rockwood 2013). Manifestations of ageing at the cellular level, and their relationship to clinically detectable age-related health changes, have been investigated by studying how clinical laboratory abnormalities change with age. Building on work from pre-clinical FI models (Feridooni et al. 2014; Miller 2017; Parks et al. 2012; Whitehead et al. 2014), we “back translated” to humans by building an FI (the FI-Lab) composed entirely of abnormal vital signs and laboratory test results (Howlett et al. 2014). This work has been multiply replicated, making clear that such deficits accumulate with age, and are “subclinical” in that commonly they are present in people with few clinically detectable health deficits (Blodgett et al. 2016; Howlett et al. 2014; Klausen et al. 2017; King et al. 2017). Their impact is non-trivial: even in those people with little evidence of frailty otherwise, increasing FI-Lab scores have been associated with a higher risk of death.

To now, such work has been done in older adults, with one report from a clinical cohort of men from late middle age (Blodgett et al. 2016). Here, in the National Health and Nutrition Examination Survey (NHANES), we aimed to use the FI-Lab to study frailty as variable deficit accumulation across the adult life course. Our main objective was to compare the FI-Lab to an FI based on clinical data; additionally, we investigated if the combination of subclinical deficits with clinical ones increased the predictive ability of the frailty index in relation to mortality.

## Methods

### Participants/setting/sample

The NHANES are a series of cross-sectional surveys that examine the health status of a nationally representative sample using examination, questionnaire and laboratory data (CDC 2014). Combining the 2003–2004 and 2005–2006 cohorts gave a total of 10,020 individuals aged 20 and older. There were no laboratory test data for 968; of the remaining 9052 people, we could not calculate the FI-Self-report (see below) for 16, the FI-Lab for 140 and the FI-Combined for 154. With 18 people missing data for one index, 136 missing data for two indices and 10 missing mortality data, the final total sample size was 8888 and included only those who had a valid FI score for all three indices. Follow-up mortality data were available through public use-linked mortality files with death certificate records from the National Death Index. All participants signed written consent forms. Ethical approval for the NHANES study was given by the Institutional Review Board of the Centers for Disease Control and Prevention (CDC 2014).

### Frailty index construction

Any FI operationalizes frailty by counting the number of deficits in an individual and dividing by the total deficits considered (Searle et al. 2008). For example, an individual with 18 of a possible 36 deficits would have an FI score of 18/36 = 0.5. A score of 0 represents full health, whereas a score of 1 represents a theoretical “complete” frailty (empirically, however, > 99% of people have FI scores < 0.7) (Howlett and Rockwood 2013). As noted, building on a previously published FI in NHANES (Blodgett et al. 2015), three separate FIs were created for the NHANES dataset. The FI-Self-report considered 36 deficits, and the FI-Lab (following earlier work by our group (Howlett et al. 2014)) identified 32 deficits from common blood and urine tests, in addition to blood pressure and pulse (Supplemental Table 1). Normal reference ranges for each variable were used to code each deficit (Blodgett et al. 2015; Henry 1991; Jones et al. 2012; Pickering et al. 2005); where applicable, sex-specific references ranges were used. Each variable was scored “1” if the value fell outside the normal range and “0” otherwise (see Supplemental Table 1). As elsewhere (Blodgett et al. 2016; Howlett et al. 2014), to create the FI-Combined, the FI-Lab and the FI-Self-report were summed, for a total of 68 items. For each FI, a frailty score was only calculated for individuals in whom < 20% of the variables were missing.

### Statistical analysis

Analyses were conducted using SPSS 20, and graphs were created using R 2.15. An alpha level of 0.05 was used to determine statistical significance. Demographic characteristics of the sample were expressed using mean FI scores ± SD. A Pearson correlation coefficient was used to examine the association between FI-Self-report and FI-Lab. Density distribution curves were created for all frailty indices. For all FIs, curve estimation was used to assess the fit of different regression models of age and frailty score. Cox regression models and AUCs respectively examined the predictive and discriminative ability of each FI and all-cause mortality. Analyses were adjusted for continuous age and sex, and the hazard ratios represent the increased odds of having the adverse health outcome for each category increase in frailty score (0–0.1, 0.1–0.2, 0.2–0.3, 0.3–0.4, 0.4+). We first examined the regressions in the full sample and then did stratum-specific analyses by age category: 20–39, 40–65 and > 65. Kaplan-Meier curves and log-rank tests demonstrated differences in mortality rate by frailty group for each index.

## Results

**Fig. 1:**
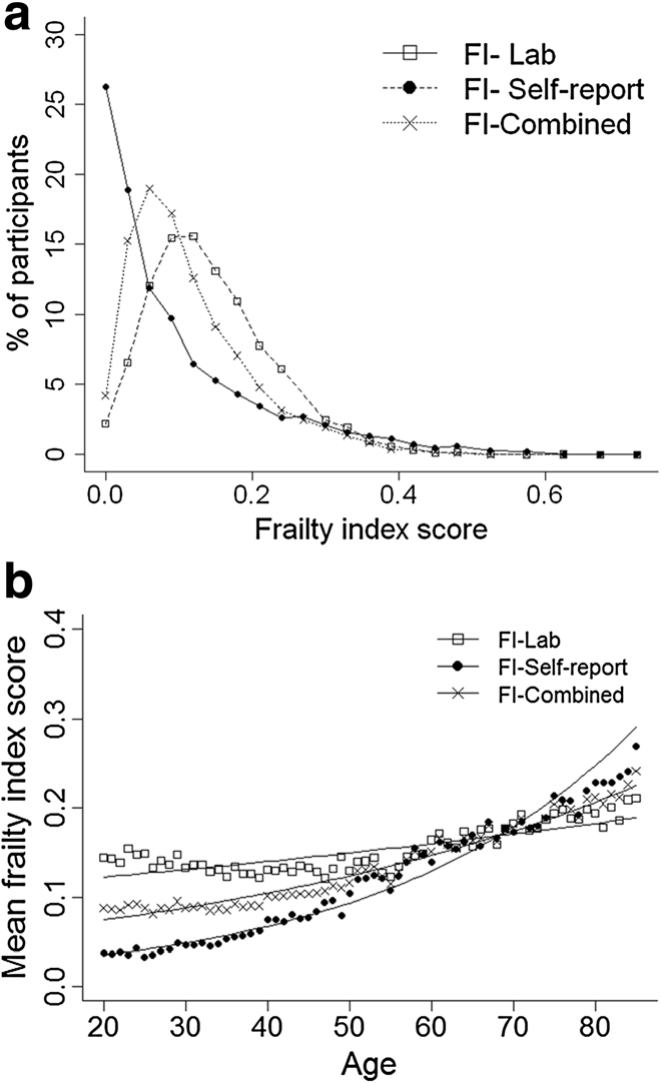
**a** Distribution by frailty index type. **b** Association between age and frailty index score (by frailty index type)

In total, 8888 participants (mean age 49.4 ± 19 years; 51.7% women, 14.1% disability as measured by activities of daily living) were included in the analyses (Table 1). Those who were excluded due to missing frailty data were slightly older (mean age 51.5 ± 21 years) and more often were women (54.7%). The Pearson correlation coefficient between the FI-Lab and the FI-Self-report was 0.33 (*p* < 0.001). All FI distributions were skewed with a long right tail, but fewer people had low levels of frailty based on the FI-Lab compared to the FI-Self - report (Fig. 1a). Mean FI scores were 0.11 ± 0.11 for FI-Self-report, 0.15 ± 0.09 for FI-Lab and 0.13 ± 0.08 for FI-Combined (Table 1). The theoretical maximum (99th percentile) for all frailty indices was well below 0.7 (FI-SR 0.49; FI-Lab 0.41; FI-Combined 0.40). Mean frailty scores increased with age non-linearly with all three indices (Fig. 1b). The slope of the natural logarithm of the FIs versus age was 0.034 (95% CI 0.033–0.035), 0.007 (95% CI 0.006–0.007) and 0.018 (95% CI 0.017–0.018) for the FI-Self-report, FI-Lab and FI-Combined respectively. The FI-Lab was higher than the FI-Self-report in those aged 20–39 (*p* < 0.001) and 40–65 (*p* < 0.001); however, the pattern reversed in those aged > 65 (*p* < 0.001) (Fig. 1b).

**Table 1:** Descriptive characteristics of the full sample and by age group

	Full sample *N* = 8888	20–39 years *N* = 3235	40–65 years *N* = 3482	> 65 years *N* = 2171
Gender [*n* (%)]*				
Men	4293 (48.3)	1463 (45.2)	1715 (49.3)	1115 (51.4)
Women	4595 (51.7)	1772 (50.8)	1767 (48.7)	1056 (51.6)
Education group [*n* (%)]*				
Less than high school	2528 (28.5)	791 (24.5)	884 (25.4)	853 (39.5)
High school	2167 (24.4)	779 (23.6)	822 (26.2)	567 (24.4)
Some college/associate degree	2476 (27.9)	1028 (31.8)	1030 (29.6)	418 (19.3)
College graduate or more	1704 (19.2)	637 (19.7)	743 (21.4)	324 (15.0)
Marital status group [*n* (%)]*				
Married	5512 (62.1)	1969 (60.9)	2361 (67.8)	1182 (54.5)
Widowed	866 (9.8)	5 (0.2)	156 (4.5)	705 (32.5)
Divorced/separated	1097 (12.3)	212 (6.6)	674 (19.4)	211 (9.7)
Never married	1407 (15.8)	1049 (32.4)	286 (8.2)	72 (3.3)
Income group [*n* (%)] *				
< $20,000	2036 (24.5)	643 (20.9)	690 (20.6)	736 (36.1)
$20,000–$45,000	2794 (33.0)	1032 (33.6)	964 (28.8)	798 (39.1)
$45,000–$75,000	1774 (21.0)	696 (22.7)	763 (22.8)	315 (15.4)
> $75,000	1817 (21.5)	700 (22.8)	926 (27.7)	191 (9.4)
Frailty score [mean ± SD]				
FI-Self-report**	0.11 ± 0.11	0.04 ± 0.05	0.11 ± 0.11	0.21 ± 0.13
FI-Lab**	0.15 ± 0.09	0.14 ± 0.08	0.14 ± 0.08	0.19 ± 0.09
FI-Combined**	0.13 ± 0.08	0.09 ± 0.05	0.13 ± 0.08	0.20 ± 0.09

*p < 0.001 (chi-squared test); **p < 0.001 (one-way ANOVA)

**Fig. 2:**
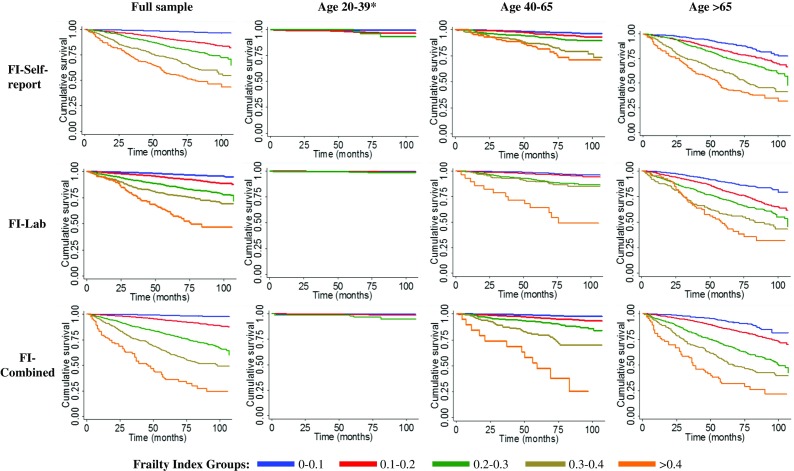
Kaplan-Meier curves showing the relationship of frailty levels with time to death

Within the follow-up period, 907 people died (10.2%). Mortality increased with age from 0.7% (23/3235) in age 20–39 to 5.7% (198/3482) in age 40–64 to 31.6% (686/2171) in age > 65 (*p* < 0.001). Absolute mortality rates increased as frailty scores increased, rising from 1.8–3.7% in the healthiest (FI < 0.1) to 48.3–69.9% in the frailest group (FI 0.4+) (Table 2). The AUCs for Fi-Self-report, FI-Lab and FI-Combined were 0.82, 0.72 and 0.83 respectively. Log-rank tests for all FIs were statistically significant (see Fig. 2; *p* < 0.001). Age- and sex-adjusted Cox regression models showed that higher levels of frailty were also associated with a higher risk of death for each FI (Table 3). Individuals in the frailest category had a much higher risk of death compared to the healthiest group, no matter which FI was used (Table 3). Both the FI-Lab and FI-Self-report remained individually significant in a combined model for death prediction (Fig. 2; model 4, Table 3). Results were similar when stratifying by age group; all associations remained significant except the 0.1–0.2 FI-Lab category for the younger and middle-aged groups.

**Table 2:** Mortality rates by frailty group category [number of deaths/number of individuals (%)]

Frailty category	FI-Self-report	FI-Lab	FI-Combined
Full sample [total: 907/8888 (10.2%)]*			
0–0.1	143/5424 (2.6%)	114/3124 (3.7%)	73/3988 (1.8%)
0.1–0.2	234/1795 (13.0%)	323/3613 (8.9%)	298/3330 (9.0%)
0.2–0.3	210/914 (23.0%)	287/1554 (18.5%)	304/1107 (27.5%)
0.3–0.4	180/475 (37.9%)	128/483 (26.5%)	174/380 (45.8%)
> 0.4	140/280 (50.0%)	55/114 (48.3%)	58/83 (69.9%)
Age 20–39 years [total: 23/3235 (0.7%)]*			
0–0.1	12/2912 (0.4%)	6/1323 (0.5%)	8/2163 (0.4%)
0.1–0.2	8/261 (3.1%)	10/1341 (1.1%)	11/975 (1.1%)
> 0.2^a^	3/62 (4.8%)	7/571 (1.2%)	4/97 (4.1%)
Age 40–65 years [total: 198/3482 (5.7%)]*			
0–0.1	61/2066 (3.0%)	41/1377 (3.0%)	31/1554 (2.0%)
0.1–0.2	45/794 (5.7%)	60/1391 (4.3%)	74/1372 (5.4%)
0.2–0.3	32/345 (9.3%)	64/534 (12.0%)	49/419 (11.7%)
0.3–0.4	38/190 (20.0%)	20/152 (13.2%)	32/118 (27.1%)
> 0.4	22/87 (25.3%)	13/28 (46.4%)	12/19 (63.2%)
Age > 65 years [total: 686/2171 (31.6%)]*			
0–0.1	70/446 (15.7%)	67/424 (15.8%)	34/271 (12.6%)
0.1–0.2	181/740 (24.5%)	253/881 (28.7%)	213/983 (21.7%)
0.2–0.3	176/523 (33.7%)	220/589 (37.4%)	252/600 (42.0%)
0.3–0.4	141/272 (51.8%)	105/211 (49.8%)	141/254 (55.5%)
> 0.4	118/190 (62.1%)	41/66 (62.1%)	46/63 (73.0%)

*p < 0.05 (chi-squared test)

^a^Frailty groups 0.2–0.3, 0.3–0.4 and > 0.4 were collapsed due to insufficient sample size

**Table 3:** Cox regression models of the association between frailty group category and time to death [hazard ratio (95% confidence interval)]

Frailty category	Model 1	Model 2	Model 3	Model 4	
	FI-Self-report	FI-Lab	FI-Combined	FI-Self-report	FI-Lab
0–0.1	Reference	Reference	Reference	Reference	Reference
Full sample^a^					
0.1–0.2	1.61 (1.29, 2.00)	1.63 (1.32, 2.03)	2.02 (1.55, 2.63)	1.50 (1.20, 1.86)	1.57 (1.26, 1.94)
0.2–0.3	2.27 (1.80, 2.86)	2.59 (2.08, 3.24)	4.02 (3.06, 5.28)	1.95 (1.55, 2.45)	2.17 (1.73, 2.71)
0.3–0.4	4.16 (3.28, 5.28)	3.62 (2.80, 4.68)	6.83 (5.09, 9.16)	3.24 (2.53, 4.13)	2.57 (1.98, 3.34)
> 0.4	6.01 (4.65, 7.77)	6.35 (4.58, 8.80)	14.50 (10.08, 20.87)	4.56 (3.50, 5.93)	3.97 (2.84, 5.54)
Age 20–39 years^b^					
0.1–0.2	7.99 (3.23, 19.74)	1.65 (0.60, 4.53)	3.48 (1.38, 8.76)	7.54 (3.05, 18.67)	1.49 (0.54, 4.12)
> 0.2^c^	12.33 (3.48, 43.74)	3.03 (1.00, 9.16)	13.87 (4.07, 47.24)	11.36 (3.19, 40.48)	2.49 (0.82, 7.54)
Age 40–65 years^b^					
0.1–0.2	2.03 (1.38, 2.99)	1.48 (0.99, 2.20)	2.87 (1.89, 4.37)	1.71 (1.16, 2.53)	1.26 (0.85, 1.88)
0.2–0.3	3.46 (2.25, 5.31)	4.29 (2.90, 6.36)	6.65 (4.24, 10.44)	2.61 (1.69, 4.06)	3.10 (2.07, 4.64)
0.3–0.4	8.15 (5.42, 12.26)	5.01 (2.93, 8.55)	17.38 (10.59, 28.53)	5.63 (3.68, 8.61)	2.77 (1.59, 4.83)
> 0.4	11.54 (7.05, 18.88)	20.91 (11.19, 39.09)	63.34 (32.28, 124.27)	7.03 (4.19, 11.80)	7.98 (4.12, 15.43)
Age > 65 years^b^					
0.1–0.2	1.69 (1.28, 2.22)	1.95 (1.49, 2.55)	1.84 (1.28, 2.64)	1.62 (1.23, 2.14)	1.82 (1.39, 2.38)
0.2–0.3	2.46 (1.87, 3.25)	2.78 (2.12, 3.66)	4.21 (2.94, 6.02)	2.20 (1.66, 2.91)	2.15 (1.63, 2.83)
0.3–0.4	4.78 (3.58, 6.37)	4.15 (3.05, 5.64)	6.80 (4.67, 9.90)	3.87 (2.88, 5.20)	2.68 (1.95, 3.67)
> 0.4	7.37 (5.46, 9.96)	5.91 (4.00, 8.73)	12.92 (8.27, 20.18)	5.77 (4.24, 7.86)	3.20 (2.14, 4.78)

^a^Age (continuous years)- and sex-adjusted models

^b^Sex-adjusted models

^c^Frailty groups 0.2–0.3, 0.3–0.4 and > 0.4 were collapsed due to insufficient sample size

## Discussion

Building on previous reports from the Canadian Study of Health and Aging (CSHA) (Howlett et al. 2014) and the European Male Ageing Study (Blodgett et al. 2016), we examined the characteristics of an FI created from 36 commonly laboratory and clinical tests, and its ability to predict mortality. The FI-Lab demonstrated some similar characteristics that are typical for self-report FIs including non-linear increase with age, submaximal limits and associations with mortality. Notably, the FI-Lab was higher than the FI-Self-report up to the age of 65, and the rate of change with age, and thereby the proportion of people with low levels of frailty, differed between the two FIs, as was also seen in an Irish report (Theou et al. 2014). Here, the association of the FI-Lab score with mortality was strongest at older ages; the evidence that higher FI-Lab scores were associated with an increased risk of death in younger individuals was limited and warrants further evaluation. In contrast to the one person in four with no self-reported deficits, less than one in 40 (2.2%) had FI-Lab scores = 0 (Fig. 1), consistent with the notion that age-related change begins subclinically. Still, the proportion with no FI-Lab deficits varied by age, from 2.3% at ages 20–39 to 2.7% at ages 40–65 and 1.3% in those greater than age 65. After age 39, the effect of adding laboratory and pulse/blood pressure measures was to lower mortality amongst those with low FI-Combined scores and increase it amongst those in whom the FI-Combined measures were high. The study also demonstrated that combining laboratory values with self-reported items increased the association of the FI with risk of death, particularly in older adults. Whether this might also hold for simpler frailty screening measures (Clegg et al. 2015) bears investigation, for example to see if adding the FI-lab to a frailty screening measure might improve sensitivity and specificity.

Studies of laboratory values as biomarkers of health and ageing typically have focused on individual test values (Darvin et al. 2014; Landi et al. 2008; Tajar et al. 2013). A previous paper (Howlett et al. 2014) demonstrated that the FI-Lab (i.e. a frailty index consisting entirely of laboratory and clinical test values) strongly predicted mortality in older adults. That study only included people aged > 65 years, and oversampled for cognitive impairment, reflected in a mean FI-Lab score of 0.27 ± 0.11, higher than scores reported here. To date, most studies on frailty have sampled people aged > 50 or 65 (Fried et al. 2001; Goggins et al. 2005; Theou et al. 2012, 2013) although one report associated frailty with mortality in people as young as 15 (Rockwood et al. 2011) and another with fracture risk from age 25 (Kennedy et al. 2014). We found significant associations between each FI and risk of death across all age strata. Of note, the hazard ratios for all FIs were significant even amongst those aged 20–65.

More studies are now investigating FIs in relation to various laboratory tests, such as those related to endocrine function, metabolism (Kim et al. 2013), inflammation (Collerton et al. 2012; Hubbard et al. 2009) and biomarkers of ageing (Canevelli et al. 2017; Hubbard et al. 2009) including DNA methylation age (Kim et al. 2017). Another body of work has investigated heritability of longevity in relation to the FI (Kim et al. 2013). Each of these settings provides a useful opportunity to test the hypothesis, arising from this study and earlier FI-Lab reports (Blodgett et al. 2016; Howlett et al. 2014), that laboratory test abnormalities indicate pre-clinical frailty. The small number of deaths and limited association between FI-Lab and mortality in those aged 20–39 indicates that mortality may not be the best outcome to assess pre-clinical and clinical frailty. Future studies should examine how the FI-Lab and FI-Self-report in younger ages are associated with other adverse health outcomes including self-reported health and healthcare utilization.

How laboratory tests fit as major and minor deficits is not clear; further investigation is required. The rate of accumulation of laboratory deficits with age was much lower although it may occur commonly in younger adults. Adding more deficits to the FI can strengthen its predictive ability (Gobbens and van Assen 2012); here, the 68-item FI-Combined showed a higher hazard ratio than did the 36-item FI-Self-report and 32-item FI-Lab. Similarly, both previous FI-Lab studies (Blodgett et al. 2016; Howlett et al. 2014) showed that combining self-reported and clinical measures increased the prediction of mortality. Whether this reflects more the number of items or their nature is not clear and requires further investigation (Gobbens and van Assen 2012; Mitnitski et al. 2017; O’Connell et al. 2015).

Our data must be interpreted with caution. Even though the 2003–2004 and 2005–2006 cohorts included over 20,000 subjects, due to age restrictions and missing laboratory data (~ 10% of those eligible), only 8888 were included here. Excluded individuals were likely older and potentially frailer. Nonetheless, our data are consistent with previous findings using a similar FI-Lab approach (Blodgett et al. 2016; Howlett et al. 2014). The cross-sectional nature of NHANES restricted the outcomes of interest to mortality, and we were unable to investigate if subclinical deficits were detectable prior to clinical ones, although the findings here (Fig. 1) suggest that this is plausible. Future research should examine the association between frailty (as measured by the FI-Lab) and other health measures, particularly in a younger sample, as well as examining temporal prevalence of frailty deficits. Clinically visible deficits must arise as a consequence of what is happening at the organ, tissue and cellular/subcellular level. Exactly how subcellular deficits scale up to become clinically visible is not yet clear. A recent proposal suggests that, in general, this reflects accumulation of damage that goes unremoved or unrepaired, and can be modelled at a systems level (Mitnitski et al. 2017). These ideas are motivating further inquiry by our group.

## Conclusions

This study sheds light on the association between pre-clinical deficits and mortality. Here, we demonstrated the usefulness of the FI-Lab in support of the concept that the deficit accumulation of ageing occurs earlier in life and has potential to assist in early identification of frailty. Whether individuals who show only laboratory and clinical test abnormalities develop frailty sooner is not yet known, but their higher mortality rates in the CSHA suggests that this is likely (Howlett et al. 2014). In conclusion, this paper establishes three key points abut population ageing: first, it suggests that even minor laboratory abnormalities, as they accumulate, increase mortality risk; second, it is a strong test of how deficits scale earlier in life to become clinically visible; and third, it greatly opens up the feasibility of expanding the content of a frailty index in routine practice.

## Electronic supplementary material

Supplemental Table 1 (DOCX 23 kb)
