# Supplementary material for: A frailty index from common clinical and laboratory tests predicts increased risk of death across the life course
Source: GeroScience. 2017 Sep 2;39(4):447–55. doi: 10.1007/s11357-017-9993-7 (PMC5636769; doi:10.1007/s11357-017-9993-7)
Supplement: Supplementary file 1 — (DOCX 23 kb) [file 11357_2017_9993_MOESM1_ESM.docx]

| **Combined Frailty Index (FI-Combined) items** | **Self-reported Frailty Index (FI-Self-report) items** | 1. Angina/ angina pectoris | 19. Difficulty walking between rooms on same floor |
| --- | --- | --- | --- |
|  |  | 2. Broken hip | 20. Arthritis |
|  |  | 3. Cancer | 21. Diabetes |
|  |  | 4. Cataract operation | 22. High blood pressure |
|  |  | 5. Confusion or inability to remember things | 23. Frequency of healthcare use |
|  |  | 6. Cough regularly | 24. General hearing |
|  |  | 7. Difficulty attending social event | 25. General vision |
|  |  | 8. Difficulty dressing yourself difficulty | 26. Health compared to 1 year ago |
|  |  | 9. Difficulty getting in and out of bed | 27. Heart attack |
|  |  | 10. Difficulty grasping/holding small objects | 28. Heart disease |
|  |  | 11. Difficulty lifting or carrying | 29. Leaked/ lost control of urine |
|  |  | 12. Difficulty managing money | 30. Medications |
|  |  | 13. Difficulty preparing meals | 31. Osteoporosis |
|  |  | 14. Difficulty pushing or pulling large objects | 32. Overnight hospital stays |
|  |  | 15. Difficulty seeing steps/curbs in dim light | 33. Self-reported health |
|  |  | 16. Difficulty standing up from armless chair | 34. Stroke |
|  |  | 17. Difficulty stooping, crouching, kneeling | 35. Thyroid condition |
|  |  | 18. Difficulty using fork and knife | 36. Weak/failing kidneys |
|  | **Laboratory Frailty Index (FI-Lab) items** | 1. Albumin (32-45 g/L) | 17. Mean arterial pressure (70-105 mmHg) |
|  |  | 2. Alkaline phosphotase (20-130 U/L) | 18. Mean cell volume (80-96 fL) |
|  |  | 3. Bicarbonate (21-28 mmol/L) | 19. Phosphorus (0.74-1.52 mmol/L) |
|  |  | 4. Bilirubin, total (2-21 umol/L) | 20. Platelet count SI (150-450 1000 cells/uL) |
|  |  | 5. Blood pressure- diastolic (60-90 mmHg) | 21. Protein, total (60-78 g/L) |
|  |  | 6. Blood pressure- systolic (90-140 mmHg) | 22. Pulse (60-99 bpm) |
|  |  | 7. Blood urea nitrogen (2.9-8.2 mmol/L) | 23. Pulse pressure (30-65 mmHg) |
|  |  | 8. C-reactive protein (0-1 mg/dL) | 24. Red cell distribution width (11.6-14.6%) |
|  |  | 9. Creatinine (M: 60-110 umol/L, W:45-90 umol/L) | 25. Segmented neutrophils percent (40-80%) |
|  |  | 10.Direct HDL-Cholesterol (1.3+mmol/L) | 26. Sodium (136-142 mmol/L) |
|  |  | 11. Folate, RBC (376-1450 nmol/L) | 27. Total calcium (2.3-2.74 mmol/L) |
|  |  | 12. Glucose, serum (3.9-6.1 mmol/L) | 28. Total Cholesterol (3.88-6.47 mmol/L) |
|  |  | 13. Glycohemoglobin levels (0-5.7%) | 29. Triglyceride (0.11-2.74 mmol/L) |
|  |  | 14. Hemoglobin (M:13.5-18 g/dL, W:12-16 g/dL) | 30. Uric acid (M:240-510 umol/L, W:160-430umol/L) |
|  |  | 15. Iron, refigerated (10.7-26.9 umol/L) | 31. Vitamin B12, serum (118-701 pmol/L) |
|  |  | 16. Lactate dehydrogenase LDH (100-190 U/L) | 32. Vitamin D (12-50 ng/mL) |

**Supplemental Table 1.** Variables included in each frailty index

Note: (M: men, W: women)
